# Impact of Learner Autonomy on the Performance in Voluntary Online Cardiac Auscultation Courses: Prospective Self-Controlled Study

**DOI:** 10.2196/78363

**Published:** 2025-11-25

**Authors:** Yudong Fang, Ligang Fang, Xue Lin

**Affiliations:** 1 Capital Medical University Beijing China; 2 Peking Union Medical College Hospital Beijing China

**Keywords:** learner autonomy, online learning, clinical skills, medical education, student engagement

## Abstract

**Background:**

Learner autonomy—the ability to self-direct and regulate learning—is a key determinant of success in online education, yet its quantifiable impact in voluntary noncredit courses remains unclear. Understanding how autonomy translates into measurable behaviors and outcomes in clinical skills training may inform more effective online learning design and learning outcomes.

**Objective:**

This study aims to quantify the association between behavioral indicators of learner autonomy and performance in a voluntary noncredit online cardiac auscultation course.

**Methods:**

We conducted a prospective, self‑controlled, single‑center study. A total of 199 registrants (n=122 physicians and n=77 medical students) were recruited via WeChat and attended four weekly 2‑hour synchronous sessions using authentic patient heart sound recordings with imaging‑based explanations. The primary outcome was the final posttraining quiz score (0-100); training effectiveness was assessed by the pre‑ to posttraining score change. The autonomy indicators were full participation (attendance at all four sessions), in‑class engagement (number of responses to brief content‑aligned prompts posed approximately every 10-15 minutes; responses recorded for participation monitoring only), and postclass review (frequency/duration of reviewing recordings and materials). Analyses included Wilcoxon signed rank tests, *χ*^2^ tests, multivariable linear regression, and receiver operating characteristic profiling of “excellent learners” (top 10% improvement).

**Results:**

Of the 199 registrants, 146 (73.4%) attended ≥1 session and 46 (23.1%) completed all sessions. Median test scores improved from 40 (IQR 20-50) to 70 (IQR 50-83; *P*<.001). Intrinsic motivation was associated with full participation (*χ*^2^_1_=4.03; *P*=.045). In multivariable models, full participation (unstandardized B=41.55, 95% CI 24.43-58.67; standardized β=0.60; *P*<.001) and in‑class engagement (B=4.79 per additional response, 95% CI 3.05-6.45; β=0.70; *P*<.001) independently predicted higher final scores (adjusted *R*^2^=0.48). Receiver operating characteristic profiling indicated that greater postclass review (recordings/materials) led to learners achieving excellent performance.

**Conclusions:**

In this voluntary online clinical skills course, showing up consistently, engaging during class, and reviewing after class—practical expressions of learner autonomy—were key correlates of short-term performance. These behaviors may be encouraged through simple, feasible course designs such as clear expectation setting, periodic interactive prompts, and structured review opportunities, which warrant prospective evaluation in future studies.

## Introduction

Online education has become an essential component of health professional learning and continuing professional development, especially since the COVID-19 pandemic [1-3]. Among factors determining online learning success, learner autonomy—the capacity to self-direct and regulate one’s learning—has been recognized as fundamental in distance education theory [4,5] and self-determination theory [6-8]. Autonomous learners set personal goals, regulate study behaviors, and reflect on their progress, achieving deeper and more durable learning [9]. However, despite extensive theoretical discussion, quantitative evidence linking measurable expressions of autonomy—such as participation, attention, and review behaviors—to concrete learning outcomes in clinical skills training remains scarce [10,11]. Understanding this relationship may help learners and educators make more strategic use of limited study time and improve learning efficiency.

For medical professionals, online education generally follows two distinct patterns. The first consists of institution-driven structured courses provided by medical schools or training organizations through online platforms, often with mandatory participation and formal assessments [12,13]. The second is learner-driven and self-directed, where participants independently choose courses aligned with their interests or professional needs, often without credit or compulsory evaluation [7,14]. Such voluntary online courses place high demands on learners’ autonomy, time management, and self-discipline. Capturing behavioral indicators of autonomy in freely accessible nonmandatory settings and examining their relationship with learning outcomes can yield insights into continuing medical education.

Cardiac auscultation remains a crucial yet challenging diagnostic skill in clinical practice [15-17]. Traditional bedside teaching is limited by the scarcity of authentic cardiac sounds and the difficulty of linking acoustic findings to underlying pathophysiology. These challenges make auscultation training well suited to online formats that can provide repeated exposure to verified recordings and integrated visual explanations. In line with this rationale, recent studies have shown that carefully designed digital modules can complement traditional instruction and improve diagnostic competence [18].

Based on these premises, we developed a voluntary noncredit online cardiac auscultation course that used authentic patient recordings, multimodal imaging, and interactive components. To examine the learner side of online success, we quantified autonomy through observable behaviors—full participation, active in-class engagement, and postclass review frequency—and evaluated how these dimensions predicted learning outcomes. This study aimed to provide quantitative evidence on how learner autonomy influences achievement in voluntary online clinical skills training.

A preliminary version of this study was shared as a preprint on Research Square [19].

## Methods

### Learner Recruitment

This was a prospective, self-controlled, single-center study that recruited doctors and medical students interested in free cardiac auscultation training through multiple channels within our hospital and affiliated medical school. The recruitment campaign was coordinated by the hospital’s Department of Medical Education and disseminated via several official WeChat groups, including those for undergraduate medical students, staff physicians, and visiting trainees. In addition, printed recruitment posters containing a QR code for registration were displayed in various teaching and clinical areas of the hospital and medical school.

Interested individuals could scan the QR code to access an online registration form. The form collected basic demographic information, participants’ prior experience with cardiac auscultation, and their motivation for enrolling in the course. Enrollment was open until the maximum capacity of the online classroom was reached, resulting in a total of 199 registrants on a first-come, first-served basis.

### Ethical Considerations

This study was approved by the Institutional Review Board of Peking Union Medical College Hospital (I-23PJ1679). During registration, all prospective participants were presented with an electronic informed consent form that described the study purpose and procedures, explained that the online teaching sessions would be recorded, and noted that anonymized data might be used for research analysis. Only those who read and electronically signed the consent form were able to complete registration and participate in the course. Participation was entirely voluntary, and no financial or material compensation was provided.

### Teaching Process

#### Teaching Contents

The cardiac sounds used in this training were authentic recordings collected from real patients over the past decade. When patients presented with abnormal heart sounds, simultaneous phonocardiographic recordings were obtained using an electronic stethoscope, and echocardiographic videos were captured to ensure precise correlation between auscultatory findings and underlying pathophysiology. Each recording was processed using acoustic analysis software to remove distortion and verify signal integrity, and all recordings were independently reviewed and validated by experienced cardiologists to confirm diagnostic accuracy.

Through this long-term systematic collection, we established a curated library of several hundred authentic cardiac sound recordings representing a broad spectrum of pathological findings. A total of 80 representative sounds were selected from this database, together with more than 30 clinical cases, 5 animations, and 100 echocardiographic clips, to form the course materials and cover the essential elements of cardiac auscultation relevant to internal medicine and clinical diagnostics.

#### Teaching Setting

All training sessions were conducted on an online teaching platform (Plaso, PLASO Network Technologies Co, Ltd) and consisted of four 2-hour live-streamed sessions held weekly. The platform supported real-time teacher-student interaction, and participants used in-ear headphones to ensure optimal sound quality.

Participants completed a 10-item cardiac sound identification test both before and after the course. Each correct answer was awarded 10 points (maximum score of 100 per test). The pre- and posttests used different sets of heart sounds that together covered all essential auscultatory findings.

During each live session, the instructor posed brief content-aligned questions approximately every 10-15 minutes. Items were randomly drawn from a preestablished question bank covering clinically relevant cardiac sounds and key conceptual checkpoints. Typical examples included “Which of the following four murmurs best represents a patent ductus arteriosus?” and “Which of the following murmurs is the most common systolic murmur?” Learners submitted responses through the platform; answers were recorded only to confirm participation and attention, not to evaluate correctness or to influence grading.

After each class, video recordings and supplementary review materials were made available on the platform for 1 month to facilitate voluntary review.

### Definition of Key Variables

#### Motivation Classification

Learning motivation was categorized as intrinsic or extrinsic according to self-determination theory, in which intrinsic motivation refers to engaging in an activity for inherent interest or enjoyment, whereas extrinsic motivation involves participating to obtain external rewards or avoid negative consequences [20]. Motivation type was determined using a single self-report item included on the WeChat recruitment page. For physicians, the question read “Are you attending this training simply because you want to master cardiac auscultation, rather than for work or promotion needs?” This wording was chosen because, in current clinical practice, cardiac auscultation is no longer indispensable for diagnosis—most structural cardiac abnormalities can be identified by echocardiography. Therefore, physicians who voluntarily return to study auscultation, often years after graduation, generally do so out of strong personal interest and professional self-expectation rather than institutional or promotion requirements. Selecting this option was classified as intrinsic motivation, whereas choosing “because it is required for work or promotion requirements” represented extrinsic motivation. For medical students, the item was simplified to two options on the registration page: “interested in learning about heart sounds” (intrinsic motivation) or “because it is needed for work or exams” (extrinsic motivation).

Participation in training was defined as attending at least one session or reviewing the provided postclass materials at least once. Full participation was defined as attending all four sessions.

#### Other Variables

Participant engagement during sessions was measured by the duration of attendance recorded by the online system and the frequency of responses to randomly posed questions.

Postclass review activities were assessed based on the frequency and duration of video reviews and the number of supplementary materials reviewed.

Total learning time included cumulative time spent attending live classes and reviewing class materials.

Training effectiveness was determined by comparing pre- and posttraining quiz scores. Learning outcomes were indicated by the final quiz scores. Participants whose score improvement exceeded the 90th percentile of all participants were classified as excellent learners.

### Statistical Analysis

Continuous variables were assessed for normality using the Shapiro-Wilk test. Normally distributed data were presented as means (SDs), while nonnormally distributed data were expressed as medians (IQRs) or median (minimum, maximum). Categorical variables were presented as frequencies and percentages. Nonnormally distributed data were analyzed using the Wilcoxon signed rank test. Differences in categorical variables were analyzed using either the *χ*^2^ test or Fisher exact test, as appropriate.

Spearman correlation coefficients and multivariate linear regression analyses were used to identify factors influencing final quiz scores. Variables with Spearman correlation coefficients (*P*<.05) were included in the multivariate regression model. Collinearity diagnostics were performed, and variables inducing collinearity were excluded. Factors associated with excellent learners were evaluated using receiver operating characteristic (ROC) curve analysis.

All statistical analyses were performed using SPSS Statistics for Windows, Version 23 (IBM Corp) and GraphPad Prism, Version 10.1.2 (GraphPad Software). Statistical significance was defined as *P*<.05.

## Results

### Overview

**Figure 1:**
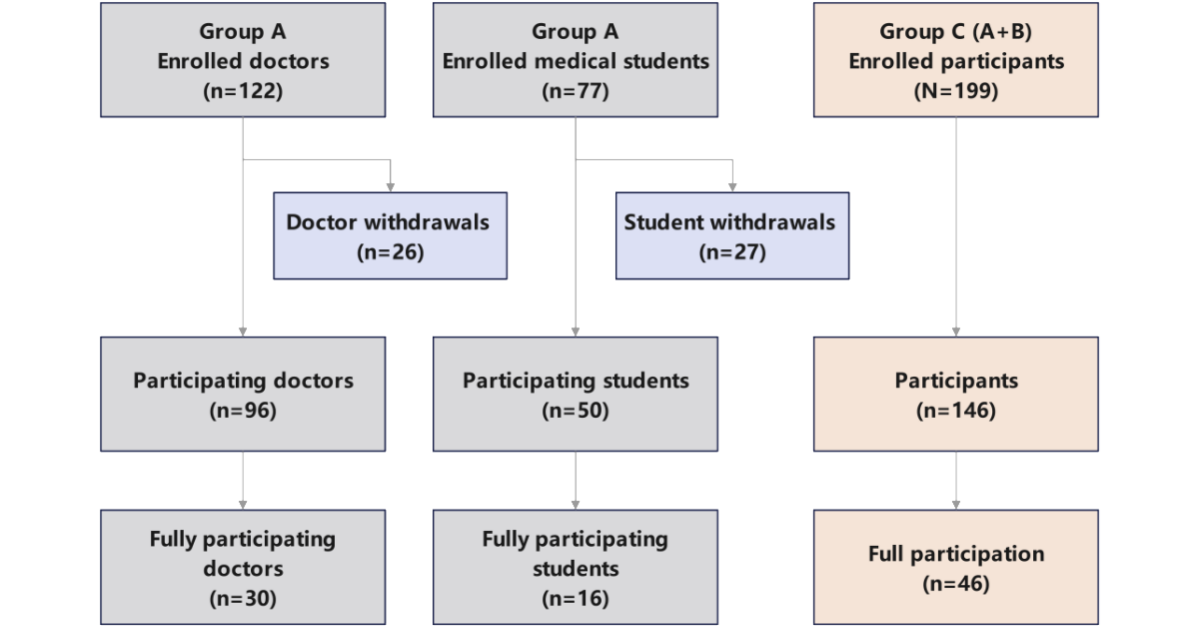
Participant flow diagram from registration to full course completion.

A total of 199 individuals voluntarily registered for the training, including 122 doctors and 77 medical students. The recruitment of doctors was completed within 2 days, whereas student recruitment required about a month. All registrants indicated that they had not yet mastered the skill of cardiac auscultation. The doctors were significantly older than the students and had substantially more years of prior exposure to auscultation learning. From registration to actual participation, a higher proportion of doctors attended the training compared with medical students (96/122, 78.7% vs 50/77, 64.9%; *χ*^2^_1_=4.57; *P*=.03). Overall, 73.4% (146/199) of registrants participated in the training, corresponding to a 26.6% (53/199) dropout rate. However, only 23.1% (46/199) of all registrants attended all four sessions (Figure 1), with no significant difference in full participation between doctors and students.

For participation motivation, doctors were more driven by intrinsic motivation than medical students (89/122, 73.0% vs 15/77, 19.5%; *χ*^2^_1_=54.10; *P*<.001). Intrinsic motivation was significantly correlated with age (*r*=0.394; *P*=.004) and total study time (*r*=0.145; *P*=.04), but not with the number of in-class random questions answered (*r*=0.153; *P*=.11). A *χ*^2^ test further indicated that intrinsic motivation was associated with full participation (*χ*^2^_1_=4.03; *P*=.045). A significant increase in posttraining scores suggested that the program was effective (Table 1).

**Table 1:** Characteristics of participants in online heart auscultation training.

Variable^a^			Total (N=199)		Doctors (n=122)		Medical students (n=77)		*P* value	
Age (years), median (IQR)			26 (23-31)		29 (26-35)		22 (20-25)		<.001	
Female sex, n (%)			136 (68.3)		91 (74.6)		49 (63.6)		.26	
Years of learning heart sound auscultation, median (IQR)			5 (3-8)		7 (5-11)		2 (1-4)		<.001	
Training participants, n (%)^b^			146 (73.4)		96 (78.7)		50 (64.9)		.03	
Participants who attended all 4 sessions, n (%)			46 (23.1)		30 (24.6)		16 (20.8)		.61	
**Motivation**										<.001
	Intrinsic motivation, n (%)	104 (52.3)		89 (73.0)		15 (19.5)				
	Extrinsic motivation, n (%)	95 (47.7)		33 (27.0)		62 (80.5)				
Pretraining score, median (IQR)			40 (20-50)		40 (20-50)		30 (20-50)		.47	
Posttraining score, median (IQR)			70 (50-83)		70 (50-90)		50 (50-75)		.29	
Individual score change, median (IQR)			30 (10-45)		35 (10-50)		30 (0-40)		.66	

^a^Continuous variables compared using the Mann-Whitney *U* test; categorical variables compared using the *χ*^2^ test or Fisher exact test, as appropriate. Two-sided *P*<.05 indicates statistical significance.

^b^Defined as participants attending ≥1 session or reviewing postclass materials at least once.

During the four sessions, a total of 16 random questions were posed, and 11 sets of review materials were distributed online after each class. The system automatically recorded the number of in-class responses and the frequency of postclass access to review materials. Of the 146 training participants, 49 (33.6%) participants watched the lecture videos after class; however, only 10 (6.8%) viewed all four full recordings. The lecture material was reviewed by 111 (76.0%) participants, but only 20 (13.7%) accessed all available materials.

The duration of postclass video viewing varied substantially among participants. Age was significantly and positively correlated with total study time (*r*=0.366; *P*<.001), time spent reviewing recorded videos (*r*=0.330; *P*<.001), and the number of review materials accessed (*r*=0.355; *P*<.001; Table 2).

**Table 2:** Assessment results for classroom attendance and postclass review. Data are limited to training attendees only.

Variable^a^	Total	Doctors	Medical students	*P* value
Live class participations (n), median (IQR)	2 (1-4)	2 (1-4)	2.5 (1-4)	.41
Total duration of live class participation (min), median (IQR)	191 (84-384)	183 (79-375)	210 (84-404)	.77
Random questions in class (n), median (IQR)	5 (1-16)	5 (1-14)	5 (3-16)	.23
Course materials reviewed after class (n), median (IQR)	1 (0-3)	1 (0-5)	0 (0-1)	.01
Duration of watching lecture videos (min), median (IQR)	0 (0-578)	0 (0-578)	0 (0-169)	.01
Total study time (min), median (IQR)	202 (86-422)	192 (86-424)	210 (88-416)	.89

^a^Continuous variables compared using the Mann-Whitney *U* test. Two-sided *P*<.05 indicates statistical significance.

### Analysis of Factors Affecting Training Scores

We performed univariate correlation analyses to examine the relationships between multiple factors—including learning motivation, overall participation, number of class attendances, duration of class participation, frequency of in-class responses to random questions, number of postclass material reviews, number of times watching lecture videos, and total study time—and the final scores. Significant correlations were observed between the final scores and full participation (*r*=0.351; *P*=.02), frequency of in-class responses (*r*=0.431; *P*=.004), and number of postclass material reviews (*r*=0.345; *P*=.03).

Age, participant type (doctor or student), years of prior auscultation study, motivation for participation, and time spent attending live sessions were not significantly correlated with final scores. Further multivariable linear regression analysis showed that only full participation and frequency of in-class responses remained independently associated with final scores, whereas postclass material review frequency did not significantly affect performance (Table 3).

**Table 3:** Multivariate linear regression results for factors associated with final auscultation scores (adjusted R^2^=0.483).

Regression variables	B (95% CI)	β	*P* value
Constant	–21.789 (–50.855 to 7.277)	—^a^	.13
Full participation	41.547 (24.426 to 58.667)	0.602	<.001
Times of answering random questions in classes	4.794 (3.054 to 6.445)	0.695	<.001

^a^Not applicable.

### Factors Influencing Becoming an Excellent Learner

**Figure 2:**
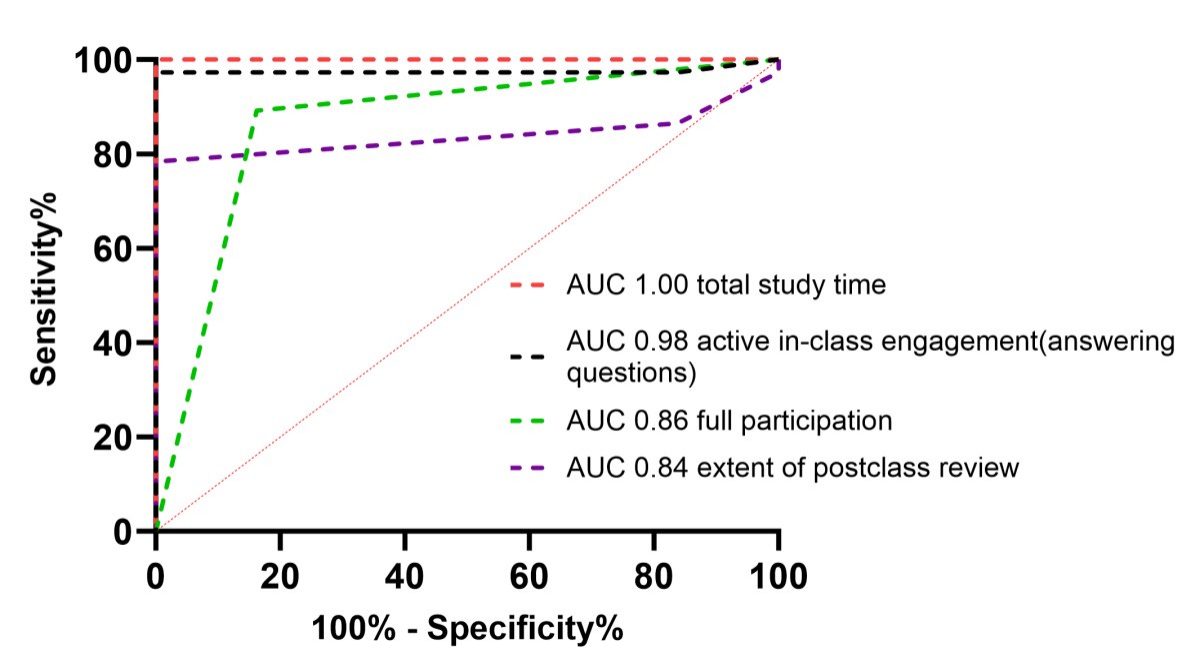
Receiver operating characteristic curve analysis of factors related to excellent performance. AUC: area under the curve.

Six participants increased their scores by more than 60 points after the training, placing them in the top 10% of all participants and defining them as excellent learners. ROC curve analysis suggests that total study time, actively answering questions in class, full participation, and the extent of postclass review were all significantly related to achieving excellent learner status (Figure 2).

## Discussion

### Principal Findings

In this voluntary noncredit online cardiac auscultation course, cohort-level test scores improved from pre- to posttraining, indicating overall instructional effectiveness. Nevertheless, only 23% of registrants completed all four sessions, underscoring the difficulty of sustaining participation in voluntary settings. Within the cohort, intrinsic motivation was positively associated with full participation, and both full participation and more frequent content‑aligned in‑class responses were independently associated with higher final scores. Postclass review was not an independent predictor in multivariable models, yet it consistently characterized learners who achieved excellent performance in ROC analyses.

### Interpretation and Comparison With Prior Work

Taken together, the data suggest a plausible pathway for success in voluntary online learning: autonomous motivation→sustained participation→active content‑aligned engagement→higher performance. This pathway is consistent with the self‑determination theory and with contemporary applications of autonomy‑supportive design in health professional education, which emphasize persistence and self‑regulation in learning tasks [20,21]. Methodologically, our study contributes by operationalizing autonomy through objective behavioral traces—attendance, periodic in‑class responses, and review—rather than relying solely on self‑report, which aligns with recent work mapping measurable dimensions of student engagement in health professional education [22].

Low completion in our cohort mirrors patterns reported in large‑scale voluntary online courses, where completion is often modest; for example, completion of massive open online courses frequently clusters around 10% in some contexts. A recent systematic review concluded that dropout is multifactorial and more closely related to course attributes, learner behaviors, and motivation/self‑regulation than to content alone, reinforcing the need for proactive planning to sustain participation in voluntary courses [11]. In practical terms, clear expectation setting before enrollment—regarding time commitment, learning objectives, and assessment schedule—and simple scheduling reminders have been reported to improve learner persistence in online education [11] and may help participants maintain full engagement in voluntary courses.

Beyond attendance, maintaining focused attention during sessions appears salient. Empirical work shows that off‑task device use is more frequent online than face‑to‑face and is associated with perceived distraction, which can erode engagement [23]. In medical education contexts, survey evidence and expert opinion further suggest that attention tends to decline after approximately 10-15 minutes unless refreshed through interactive elements. Accordingly, periodic content-aligned checks (brief questions or polls), as implemented in our study, are advisable in synchronous sessions [24].

Finally, postclass review may play a selective role in mastery. Although postclass review was not an independent predictor in our adjusted model, high performers engaged in postclass review more often. This pattern is consonant with meta-analytic evidence that spaced or retrieval-based reviews improve long-term retention and, in some cases, clinical behavior change relative to massed learning [25]. In health professional education, spaced e‑learning has been shown to enhance knowledge retention in basic life support compared with single‑session learning [26]. Future implementations could explore embedding simple, low-burden review structures—such as a 48-hour recap and a 1-week revisit—and providing automated reminders to prompt learners to consolidate key material.

Notably in our cohort, the associations between participation/engagement and performance were not explained by measured demographic characteristics (age, years of exposure to auscultation, workplace), suggesting that the behavioral pathway outlined above could be applicable across subgroups in similar settings.

### Limitations

This study has several limitations. It was conducted at a single center with voluntary participation, which may limit generalizability and introduce self-selection bias. The primary outcome was a short-term test score, and long-term retention was not assessed. Although the pre- and posttests followed the same blueprint, using different item sets may have introduced measurement variability. Motivation was assessed by a single unvalidated item, and the voluntary single‑center sample entails self‑selection; hence, residual confounding and limited generalizability remain.

### Conclusions and Broader Implications

Learners who consistently attended all sessions, stayed focused during class, and reviewed material after class achieved the best results in this voluntary online clinical skills course. These behaviors represent practical expressions of learner autonomy and can be strengthened through simple habits—committing to scheduled sessions, engaging actively with class tasks, and revisiting key materials on a spaced schedule. For busy health professionals, such disciplined learning routines may transform limited study time into durable competence.

Beyond this specific course, these findings highlight the importance of quantifying and supporting learner autonomy as a measurable construct in online medical education. Incorporating behavioral analytics to monitor participation, engagement, and review patterns may provide educators with actionable insights to design more autonomy-supportive learning environments. Future research should investigate how these learner-driven behaviors can be further supported through course design and motivation-enhancing strategies to sustain long-term engagement and performance across diverse educational settings.

## Abbreviations

ROC: receiver operating characteristic

## Data Availability

The datasets generated and analyzed during this study are not publicly available because they contain identifiable personal information and course platform activity records in Chinese. Deidentified data may be made available from the corresponding author upon reasonable request.
